# Identification of lncRNAs in peripheral blood mononuclear cells associated with sepsis immunosuppression based on weighted gene co-expression network analysis

**DOI:** 10.1186/s41065-025-00400-z

**Published:** 2025-04-07

**Authors:** Wenjia Zhang, Yan Li, Gang Li, Aijia Zhang, Wende Sun

**Affiliations:** 1 Department of Emergency Medicine, China–Japan Friendship Hospital, No.2, Yinghua Rd., Chaoyang District, Beijing, China; 2 Department of Nephrology, Jilin Province People’s Hospital, Changchun, 130022 China; 3 Department of Orthopedic and Joint Surgery, Traditional Chinese Medicine Hospital of Juxian, Rizhao, 276500 China

**Keywords:** Sepsis, Immunosuppression, lncRNAs, WGCNA, Hub genes

## Abstract

**Background:**

Sepsis-induced immunosuppression involves complex molecular mechanisms, including dysregulated long noncoding RNAs (lncRNAs), which remain poorly understood.

**Objective:**

We aimed to identify immunosuppression-related lncRNAs and their functional pathways in sepsis. Methods: Using weighted gene coexpression network analysis (WGCNA), we analyzed lncRNA profiles from peripheral blood mononuclear cells (PBMCs) of three sepsis patients and three healthy controls. Key modules linked to immunosuppression were validated via RT-PCR and external datasets. Pathway enrichment and protein interaction networks were employed to prioritize mechanisms.

**Results:**

A sepsis-associated module containing 4,193 lncRNAs revealed three immunosuppression-related pathways: Th17 cell differentiation, cytokine–cytokine receptor interactions, and cancer-related proteoglycan signaling. Protein–protein interaction networks identified three central genes (*SLFN12*, *ICOS*, *IKZF2*) and their linked lncRNAs (ENSG00000267074, lnc-ICOSLG-1, lnc-IKZF2-7), all significantly downregulated in sepsis patients.

**Conclusion:**

Our findings highlight novel lncRNA-regulated pathways in sepsis-induced immunosuppression, providing potential targets for improved diagnosis and therapy.

**Supplementary Information:**

The online version contains supplementary material available at 10.1186/s41065-025-00400-z.

## Introduction

Sepsis is a severe medical condition characterized by life-threatening organ dysfunction that results from an imbalanced host response to infection. Nearly 50 million individuals are affected by sepsis worldwide each year, with a mortality rate of approximately 22% [1]. The initial acute reaction of the host to an invading pathogen frequently triggers an overwhelming release of cytokines, often referred to as a “cytokine storm,” which leads to immunosuppression [2]. A significant number of sepsis patients succumb during the immunosuppression phase. This postsepsis immunosuppression is commonly characterized as a compensatory anti-inflammatory response syndrome [3], but its mechanisms remain unclear. Consequently, delving into the fundamental mechanisms and identifying biomarkers associated with sepsis-induced immunosuppression is important.

Long noncoding RNAs (lncRNAs) represent a class of nonprotein-coding transcripts that exceed 200 nucleotides in length [4]. Studies suggest that lncRNAs play an important role in the progression of sepsis-induced immunosuppression [5, 6]. In earlier studies, differential expression analysis was used to identify differentially expressed lncRNAs (DElncRNAs) by comparing RNA sequencing results between a disease and control groups in a rat model [7]. This approach primarily emphasizes the function of individual genes, but does not delineate the intricate relationships between genes and diseases.

A weighted gene co-expression network analysis (WGCNA) offers a solution to this limitation. WGCNA is a bioinformatics technique designed to process microarray or RNAseq data and categorize genes into distinct coexpression modules [8]. One of its strengths is the ability to transform gene expression data into coexpression modules, which offers insight into genes that are relevant to specific phenotypic features at a systemic level [9]. WGCNA is gaining traction because of its capacity to delineate the interrelationships among networks, genes, and phenotypes. It retains high sensitivity to genes with low abundance or minimal fold-changes, offering a comprehensive view without compromising data integrity [10]. Previous studies indicate that WGCNA not only highlights important modules and pathways associated with various diseases, but also identifies lncRNAs [11–13]. In a study conducted by Zhang et al. [4], four lncRNAs (GSEC, NONHSAT160878.1, XR_926068.1, and RARA-AS1) were identified as hub genes in pediatric sepsis using WGCNA. Simultaneously, another study identified differentially expressed genes present in the blood of sepsis patients, which provided insight into the associated molecular pathways [14]. To delve deeper into the immunosuppression phenotype caused by sepsis in adults, we collected PBMC samples from mature individuals.

In this study, we aimed to identify lncRNAs and pathways related to adult sepsis-induced immunosuppression using both WGCNA and the Kyoto Encyclopedia of Genes and Genomes (KEGG) pathway enrichment analysis. The results provide insight into potential pivotal genes and shed light on the underlying pathological mechanisms of sepsis immunosuppression. Furthermore, by elucidating these mechanisms, this study reveals new biomarkers for the diagnosis of sepsis, which may lead to more effective therapeutic strategies and improve sepsis management and patient outcomes.

## Material and methods

### Study design and population selection

From January 2020 to December 2022, we enrolled 23 patients with sepsis-induced immunosuppression and 13 age- and sex-matched healthy controls from the China–Japan Friendship Hospital. A statistical analysis was performed using an independent t-test for age comparison and a Chi-square test to compare sex distribution (Supplementary Table 1). There was no significant difference in the mean age between the sepsis group (49.6 ± 19.4 years) and the control group (53.5 ± 18.7 years; *p* > 0.05). Similarly, sex did not significantly differ between the sepsis (43.5% male, 56.5% female) and control groups (38.5% male, 61.5% female; *p* > 0.05), indicating a well-matched control group for age and sex. To qualify for the clinical research group, patients had to meet the following criteria:

- Adherence to the sepsis 3.0 diagnostic criteria
- Diagnosis of sepsis within 48 h
- Lymphocyte count and percentage below the standard lower limit (with the absolute lymphocyte count threshold being 1.1 × 10.^9^/L and the lymphocyte-to-white blood cell ratio threshold set at 20% at our institution)
- Age ranging from 18 to 80 years

Patients diagnosed with tumors, autoimmune disorders, hematological diseases, AIDS, or tuberculosis infections were excluded from this study. Each participant was informed of the purpose of the experiment prior to its commencement and was clearly made aware that their data would be used solely for scientific research. After fully understanding the details of the experiment, participants voluntarily signed the informed consent form. The Human Ethics Committee of the China–Japan Friendship Hospital approved the procedures in this study. The ethical approval document number for this research is zryhyy61-21–03–45.

### Sample preparation and PBMC collection

Upon admission, 5 ml of blood samples were promptly collected from individuals in both the sepsis immunosuppression and control groups. These samples were preserved in tubes containing ethylenediaminetetraacetic acid (EDTA). Peripheral blood mononuclear cells (PBMCs) were extracted from the peripheral venous blood samples of both the sepsis immunosuppression patients and healthy controls.

To isolate PBMCs, the Ficoll density gradient centrifugation method was employed. Initially, 5 ml of whole blood was drawn into an EDTA tube and subsequently transferred to a 50-ml centrifuge tube. This blood was diluted with an equal volume of phosphate-buffered saline (PBS) and mixed gently. Two 15-ml centrifuge tubes were prepared each filled with 5 ml of Ficoll solution. The diluted blood (5 ml in volume) was carefully layered over the Ficoll solution in each tube, ensuring no mixing of the two solutions. The tubes were centrifuged at 2,000 rpm for 20 min. After centrifugation, the PBMC layer, situated at the interface between the plasma and Ficoll was carefully retrieved using a sterile Pasteur pipette and transferred to a new centrifuge tube. The cells underwent two washes with PBS, with each wash involving centrifugation at 1,500 rpm for 10 min at room temperature without applying a brake. Finally, the PBMCs were resuspended in RPMI 1640 medium (Gibco, USA), which was supplemented with 10% fetal bovine serum (Sigma-Aldrich, Germany) and 1% penicillin–streptomycin (Thermo Fisher Scientific, USA).

### RNA microarray research

Total RNA was extracted from PBMCs using TRIzol reagent (Thermo Fisher Scientific) and adhering strictly to the manufacturer’s protocol. Quantification of total RNA was done using the NanoDrop ND-2000 (Thermo Scientific), resulting in a concentration of 100 ng/µL, whereas RNA integrity was evaluated with an Agilent Bioanalyzer 2100 (Agilent Technologies). RNA integrity number values spanned from 7.0 to 9.8 across all samples and the RNA quality was considered satisfactory. The processes of sample labeling, microarray hybridization, and washing were performed based on the manufacturer’s instructions. The total RNA was reverse-transcribed into double-stranded cDNA followed by synthesis into cRNA. It was then labeled with Cyanine-3-CTP. The labeled cRNAs underwent hybridization on the microarray. After washing, the arrays were analyzed using an Agilent Scanner G2505C (Agilent Technologies). The software, Feature Extraction 10.7.1.1 (Agilent Technologies), facilitated the analysis of the array images to extract the raw data. For this experiment, the Agilent Human ceRNA Microarray 2019 (4*180k, Design ID:086188) was used and comprehensive data analysis for the six samples was performed by OE Biotechnology Co., Ltd. (Shanghai, China).

### WGCNA construction and module identification

The coexpression network was constructed using the “WGCNA” package in R. Initially, the samples underwent clustering to detect any potential outliers. Subsequently, the soft-thresholding power β was determined using the “pickSoftThreshold” function in R. With the soft-thresholding power β set at 12 and ensuring an approximate scale-free topology, we proceeded with a one-step network construction and module detection. Next, we computed the module eigentones, module membership (MM), and gene significance (GS) to associate the modules with clinical characteristics. The relevant gene information for each module was extracted for an in-depth analysis. Based on the MM and GS values, the top 20 lncRNAs were identified.

### KEGG pathway enrichment analysis

We conducted a KEGG pathway enrichment analysis using the OmicShare tool, a web-based platform dedicated to data analysis (https://www.omicshare.com/tools). The results of the enriched pathways were visually represented in a bubble chart.

### PPI network

We used the STRING database (http://string-db.org) to establish a PPI network, setting a confidence score > 0.4 as the significance threshold. The target genes associated with the top 20 lncRNAs were selected and depicted using Cytoscape 3.6.0. Within this network, hub genes were identified based on their degree. Notably, genes with a high degree were considered to play a significant role in sepsis immunosuppression.

### Validation by qPCR

Total RNA was extracted from the myocardial tissue and PBMCs using TRIzol® reagent (Invitrogen, Carlsbad, CA, USA) and cDNA was synthesized using the High-Capacity cDNA Reverse Transcription Kit (Applied Biosystems, Foster City, CA, USA) based on the manufacturer’s protocol. RT-PCR was done using a SYBR Green PCR Master Mix (Applied Biosystems). The specific primers used for RT-PCR are listed in Supplementary Table 9. The relative RNA expression levels of key genes were normalized to GAPDH using the 2^−ΔΔCt^ method.

In the initial phase of the study involving lncRNA microarray analysis, three samples were included in the experimental group and three were used for the control group. For subsequent validation by quantitative PCR, the sample size was expanded to include 23 samples in the experimental group and 13 samples in the control group.

## Results

### WGCNA

**Fig. 1:**
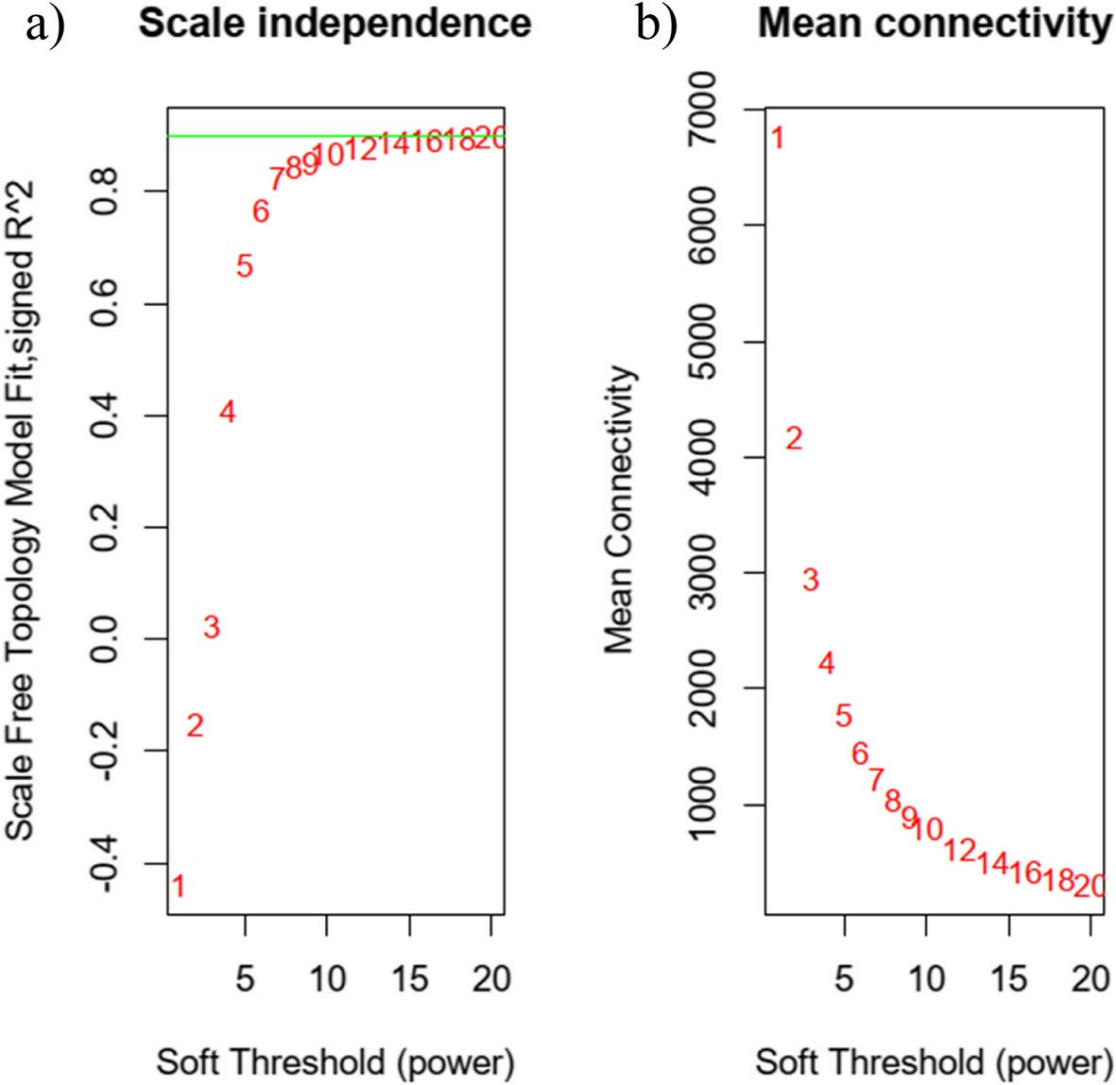
Analysis of network topology for different soft‐threshold powers. **a** The left panel illustrates the effect of soft‐threshold power on the scale‐free topology fit index. **b** The right panel shows the impact of soft‐threshold power on the mean connectivity

**Fig. 2:**
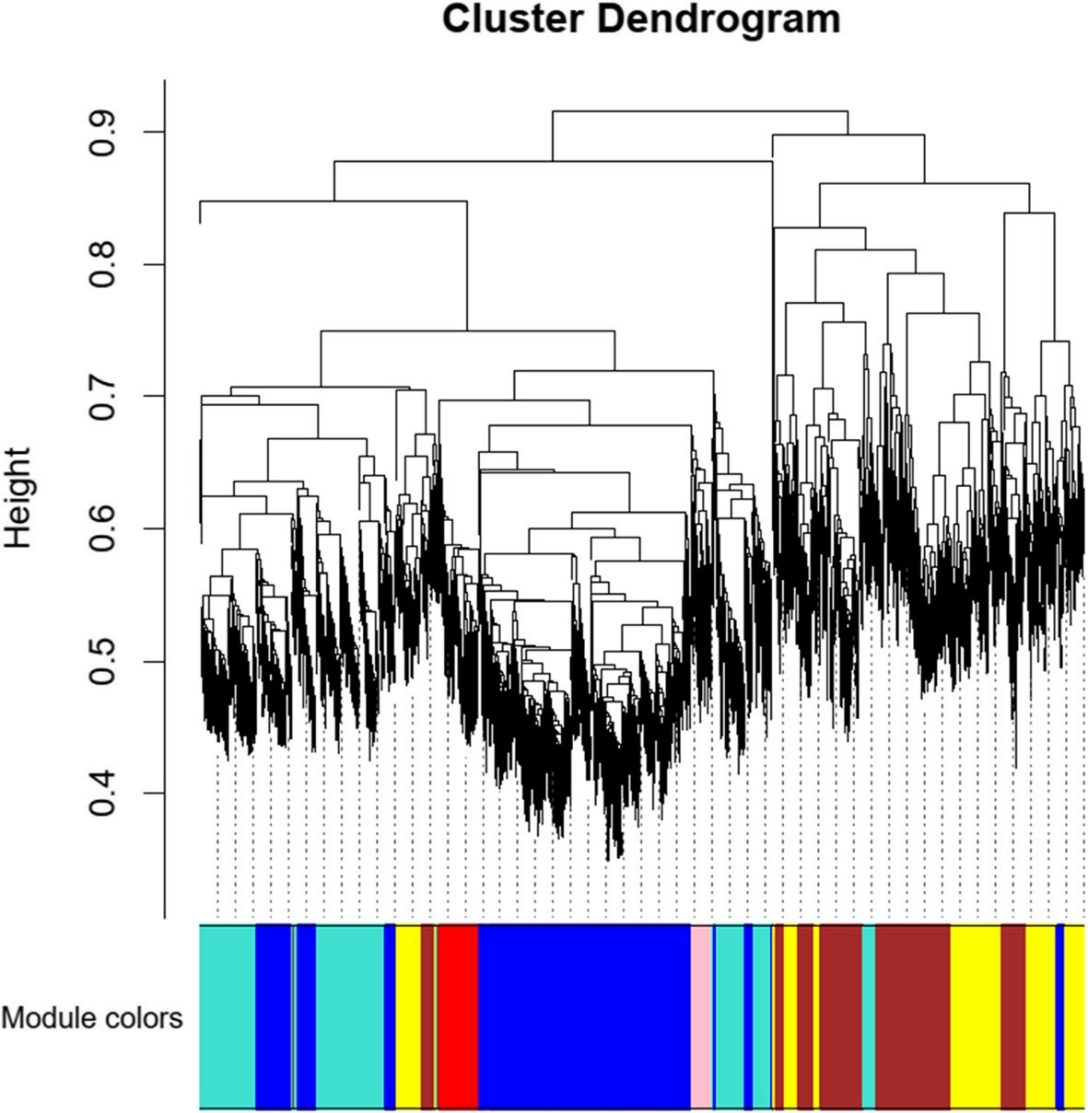
Gene clustering tree (dendrogram) obtained by hierarchical clustering of adjacency-based dissimilarity. This dendrogram represents the hierarchical clustering of genes based on their adjacency-based dissimilarity

Initially, 4193 lncRNAs were identified based on a *p*-value < 0.05 and a fold-change > 1 and subjected to WGCNA analysis. Notably, no lncRNAs were excluded either because of outliers or subsequent filtering. With a soft-threshold power set at 12, the scale-free topology index reached 0.9, as shown in Fig. 1. This configuration indicated that the network adhered to a power-law distribution, closely mirroring the genuine biological network state [15]. Figure 2 shows the gene dendrograms along with their corresponding module colors. A detailed breakdown of the number of lncRNAs within each module is provided in Supplementary Table 2.

### Identifying the key clinically significant modules

**Fig. 3:**
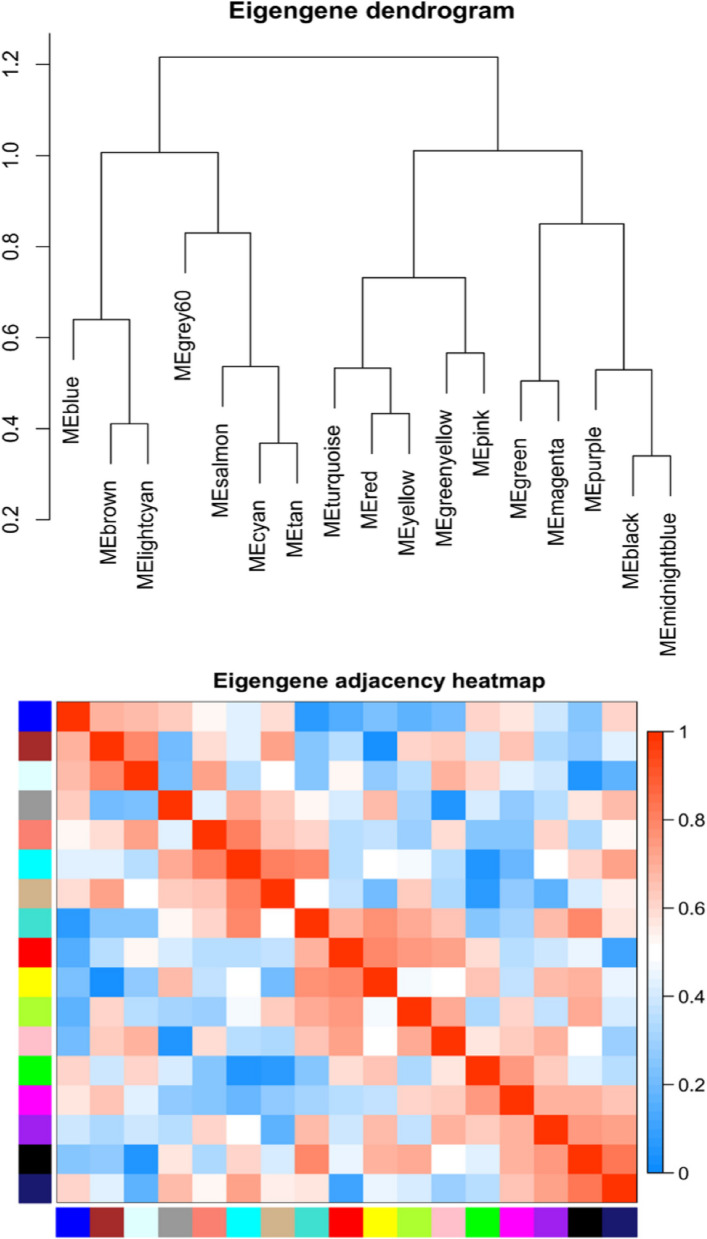
Heatmap of eigengene adjacency. The heatmap displays the adjacency relationships among eigengenes, with color bars on the left and bottom indicating the modules for each row or column

**Fig. 4:**
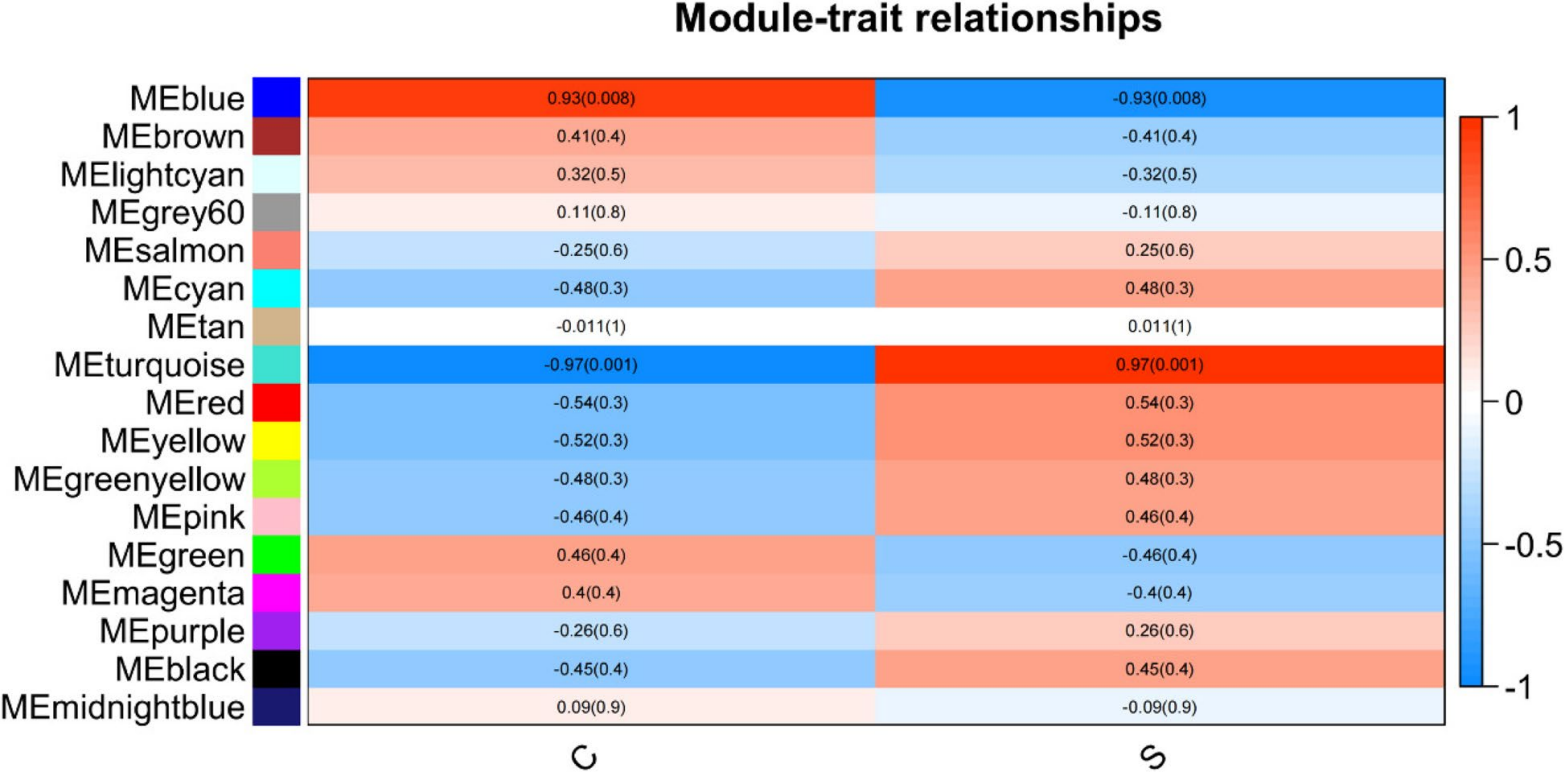
Module-trait relationships. Each row corresponds to a module eigengene, and each column corresponds to a trait (C, healthy control; S, sepsis immunosuppression). Each cell contains the corresponding correlation coefficient and *p*-value, with red indicating positive correlation and blue indicating negative correlation

The heatmap in Fig. 3 provides insight into the eigentone adjacency across various modules. Because we aimed to understand the mechanism behind sepsis immunosuppression, we incorporated data from six distinct samples: three from healthy controls and three from individuals with sepsis immunosuppression. Our primary focus was directed toward the turquoise module, which exhibited the strongest correlation (*r* = 0.97, *p* < 0.01) with the clinical characteristics of patients experiencing sepsis immunosuppression (Fig. 4).

### Hub gene identification and functional annotation

**Fig. 5:**
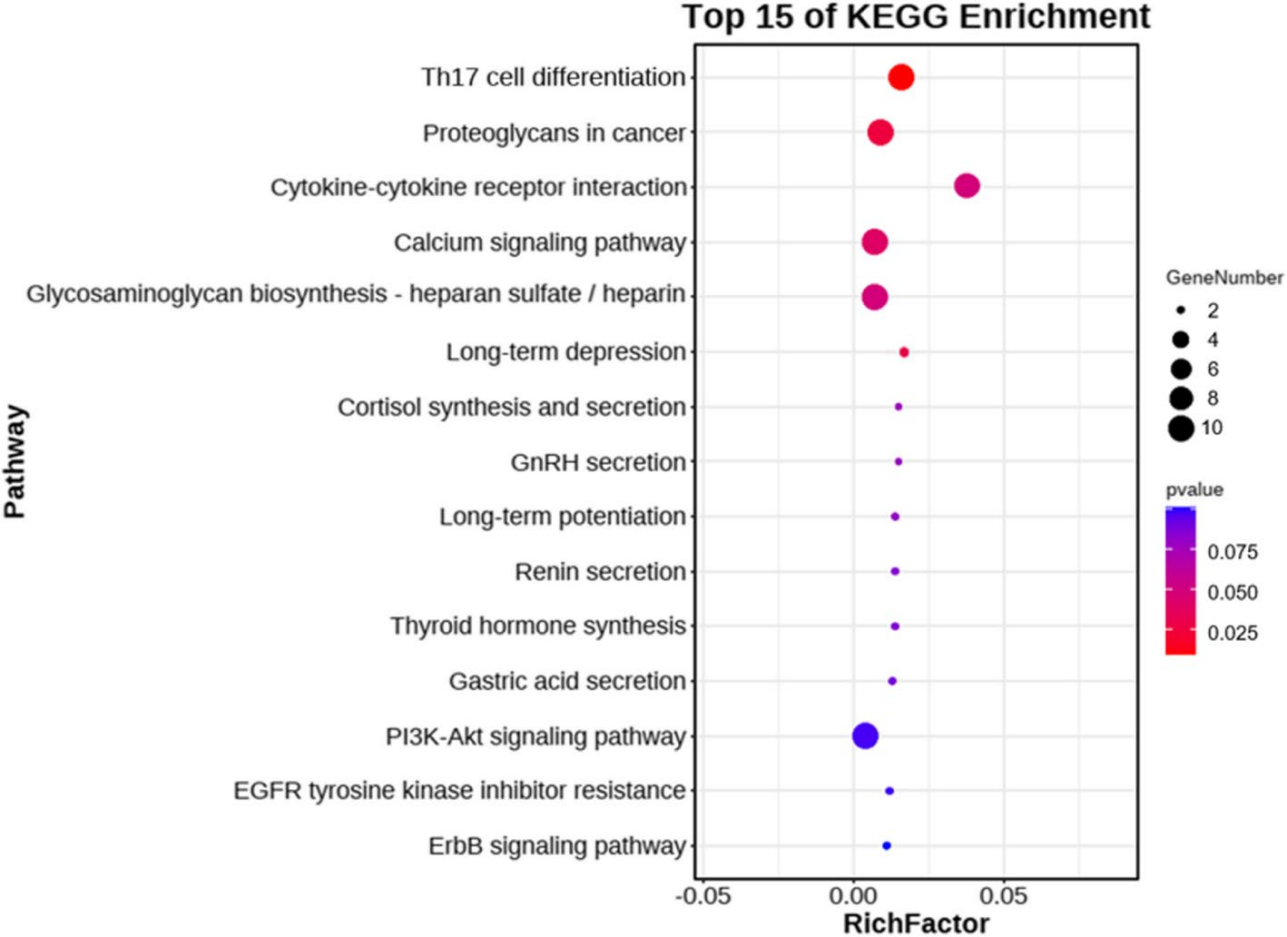
KEGG pathways enriched by the top 20 lncRNAs in sepsis immunosuppression patients. The X-axis represents the enrichment factor, and the Y-axis lists the KEGG pathways. The figure highlights the most significant pathways associated with the identified lncRNAs

From the analysis, the top 20 lncRNAs, characterized by elevated MM and GS, were earmarked for further examination, as detailed in Supplementary Table 3. The 64 target genes associated with these top 20 lncRNAs (Supplementary Table 4) within the turquoise module were processed using OmicShare. This was done to identify the most representative pathways in the KEGG database and to discern their functional attributes. The three predominant pathways identified were Th17 cell differentiation (ko04659), proteoglycans in cancer (ko0520), and cytokine–cytokine receptor interaction (ko04060), as shown in Fig. 5 and further elaborated in Supplementary Table 5.

### PPI network

**Fig. 6:**
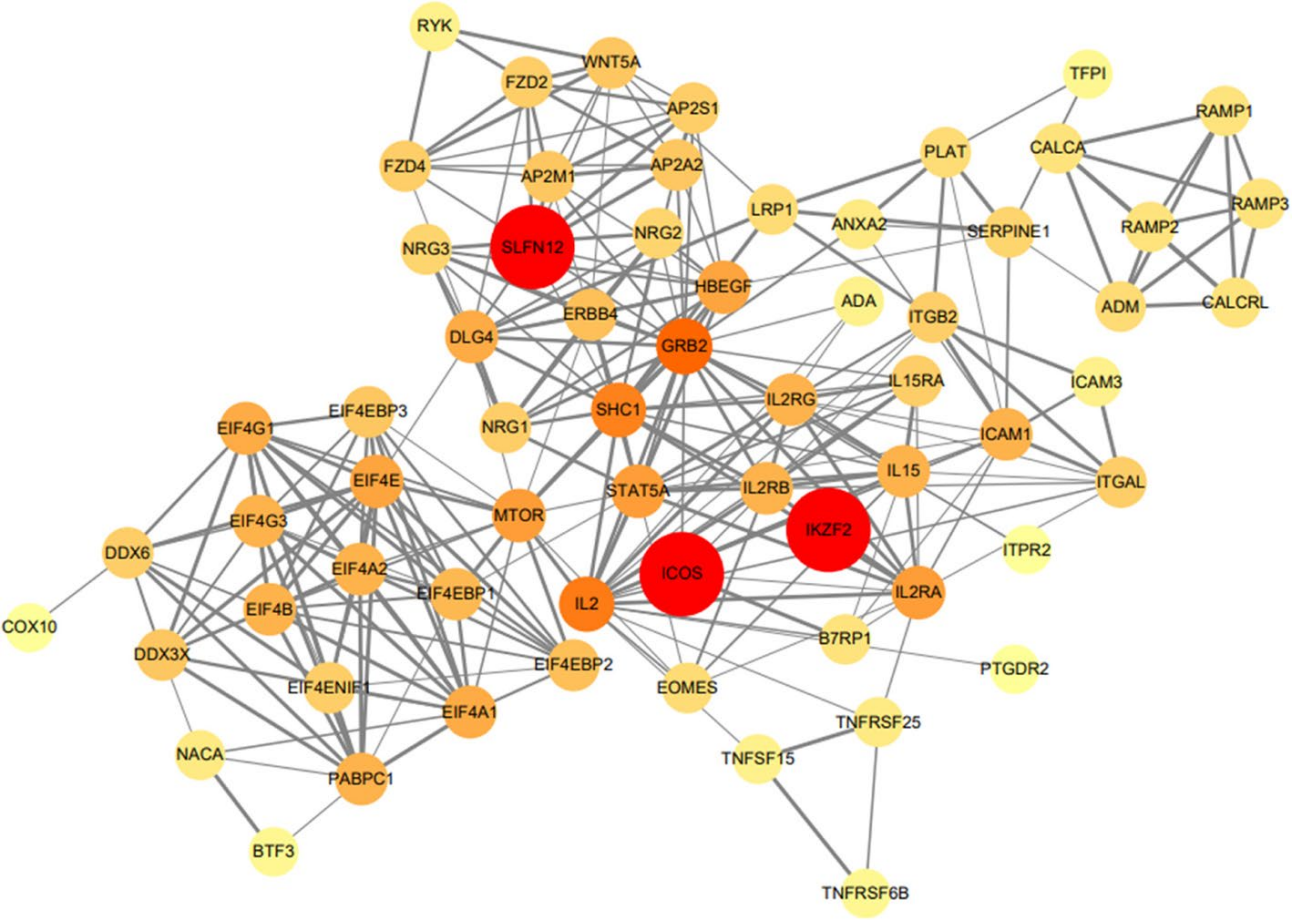
Protein–protein interaction (PPI) network and hub genes. Hub genes were identified from the top 20 lncRNAs using degree analysis. The depth of color indicates the ranking of hub genes, from low to high

Using the STRING database, we constructed a protein–protein interaction (PPI) network based on the 64 target genes previously mentioned. This network consisted of 64 nodes and 271 edges, representing proteins and their interactions, respectively. The detailed architecture of the network is shown in Fig. 6. A confidence score greater than 0.4 was established as the benchmark for significance. From this analysis, genes that ranked in the top three degrees, namely SLFN12, ICOS, and IKZF2, were identified as the central hub genes, as detailed in Supplementary Table 6.

### Validation by quantitative real-time PCR (qPCR)

**Fig. 7:**
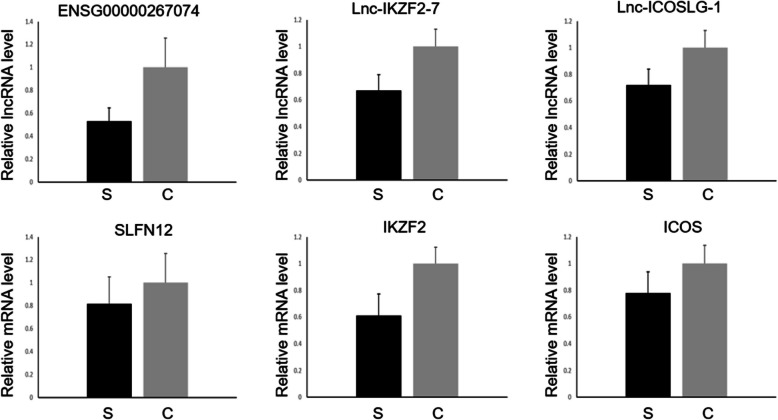
Validation of three lncRNAs by qPCR. The expression differences of three lncRNAs related to the hub genes identified by the PPI network were validated by qPCR. The figure shows the relative expression levels in sepsis immunosuppression (S) and healthy controls (C). Statistical significance is indicated as *p < 0.05, **p < 0.01, and ***p < 0.001

Validation of the expression profiles was conducted for three long noncoding RNAs (lncRNAs): ENSG00000267074, lnc-ICOSLG-1, and lnc-IKZF2-7, and three messenger RNAs (mRNAs): SLFN12, ICOS, and IKZF2. Figure 7 shows the *p*-values and relative RNA levels, revealing a marked decrease in their expression in patients suffering from sepsis-induced immunosuppression compared with healthy individuals. This marked reduction in expression suggests that these RNAs may play an important role in the immunosuppressive condition triggered by sepsis. Furthermore, the results suggest that these RNAs may serve as biomarkers for the diagnosis and treatment of the immunosuppressive state in sepsis, offering new targets for clinical intervention and management of this condition.

### Validation in the GEO data set

Because WGCNA typically requires a more extensive sample set and our study consisted of only six samples, we enhanced the accuracy and reproducibility of our experiment. To achieve this, we referenced a dataset containing lncRNA from healthy human peripheral blood single nucleated cells, which was sourced from https://www.ncbi.nlm.nih.gov/geo/query/acc.cgi?acc=GSE201958, to serve as a control.

We validated the expression patterns of ten differentially expressed lncRNAs (DElncRNAs) using the GSE201958 dataset. It is worth noting that the GSE201958 dataset was derived from the GPL20301 platform. The GPL20301 Illumina HiSeq 4000 (Homo sapiens) array encompasses the expression profiles of potential mRNAs and DElncRNAs, all of which were gauged based on probe intensity. A significant portion of the probes in this array were attributed to mRNAs, with only a limited number associated with lncRNAs.

Consequently, the lncRNA probes in this array were not sufficiently exhaustive to detect all of the targets pertinent to our study. It should be noted that studies conducting lncRNA microarray analysis in human peripheral mononuclear cells related to sepsis are scarce. The GSE201958 dataset represents the only available resource that includes lncRNAs, making it the most suitable option for the goals of our study. In this dataset, only 10 DElncRNAs from the turquoise module were identified. The associated *p*-value and log2 fold-change details are listed in Supplementary Table 7.

In addition, we conducted a comparative analysis of three pivotal genes (SLFN12, ICOS, and IKZF2) between our dataset and the GSE201958 dataset (detailed in Supplementary Table 8). The results indicated no significant differences in the expression of these genes between the two datasets. This consistency in the gene expression profiles further reinforces the validity and utility of our data and provides a robust foundation for subsequent in-depth analyses.

## Discussion

The present study focused on the role of PBMCs in sepsis immunosuppression, because they have a more pronounced contribution to immunity compared with granulocytes. Thus, we deliberately excluded granulocytes from our analysis and concentrated solely on PBMCs. It is worth noting that a prior study by Chen C et al. [14] included white blood cells as well as granulocytes; however, our objective is to explore alternative methodologies to better understand and address sepsis immunosuppression.

In this study, we used the WGCNA approach to identify pivotal lncRNAs and delve into their associated pathways and target genes in the context of sepsis immunosuppression. The three most prominent pathways were Th17 cell differentiation, proteoglycans in cancer, and cytokine–cytokine receptor interaction. Among the target genes, SLFN12, ICOS, and IKZF2 were considered particularly significant. The corresponding lncRNAs associated with the three hub genes were identified as ENSG00000267074, lnc-ICOSLG-1, and lnc-IKZF2-7.

Previous studies have shed light on the potential mechanisms underlying the three pivotal lncRNAs: ENSG00000267074, lnc-ICOSLG-1, and lnc-IKZF2-7. Notably, ENSG00000267074, a member of the Schlafen family, is associated with immune infiltration [16]. In addition, lnc-ICOSLG-1, which is part of the B7 family, has a role in modulating the immune response and acts as an immune checkpoint [17]. Furthermore, lnc-IKZF2-7 was correlated with Helios. Notably, decreased expression of Helios has been linked to the destabilization of Treg cells [18]. Delving deeper into the distinct functions and mechanisms of these lncRNAs may offer insight into their potential contributions to sepsis immunosuppression.

Th17 cells were discovered in 2003 and secrete IL-17 or IL-17A [19]. Along with Th1, Th2, and Treg cells, there are four CD4 + T-cell subsets [20]. They are differentiated by Th0 cells via IL-6 and TGF-β stimulation. The Th17 cell differentiation pathway plays an important role in immune regulation, host defense, and autoimmune diseases [21]. During sepsis immunosuppression, Th17 cells are upregulated by lactate [22]. STAT5 downregulation attenuates glucose uptake and glycolysis of Th17 cells under low-glucose/high-lactate conditions, which increases the immunosuppressive state [23].

Proteoglycans are central to the modulation of critical cellular pathways, such as inflammation and autophagy. They are deeply intertwined with both cancer initiation and sepsis immunosuppression. Notably, the removal of lumican genes, which are fundamental proteoglycans in the extracellular matrix (ECM), decreases the ability of mice to overcome polymicrobial sepsis by inhibiting the endosomal induction of type I interferons [24].

Cytokines are soluble extracellular proteins or glycoproteins that serve as vital intercellular mediators and activators of cells involved in the innate and adaptive immune responses. The interaction pathway between cytokines and their receptors plays an important role in sepsis immunosuppression [25]. Chen et al. found that the IL-10/TNF cytokine ratio is positively correlated with sepsis immunosuppression [26].

KEGG and PPI analyses highlighted the central role of SLFN12 in the onset of sepsis immunosuppression, particularly through the Th17 cell differentiation pathway. This pathway may represent a key underlying mechanism in sepsis immunosuppression. SLFN12 is part of a vertebrate gene family that encodes proteins with significant sequence similarity. These proteins are implicated in a variety of cellular activities and tissue-specific functions, including DNA replication, cell proliferation, immune and interferon responses, viral infections, and susceptibility to DNA-targeted anticancer drugs [27]. ENSG00000267074 is a newly identified lncRNA and a sense intronic lncRNA of SLFN12L described on the Genecards website. Overall, ENSG00000267074 may affect sepsis immunosuppression through the Th17 cell differentiation pathway.

In the present study, ICOS was enriched in Th17 cell differentiation and proteoglycans in cancer pathways. Wu et al. found that ICOS participates in T-cell activation and migration [28]. Moreover, ICOS surface expression is increased on T_FH_ cells from B7-H1 − / − mice in a hyperimmune state [29]. A reduction in ICOS expression may influence the progression of sepsis immunosuppression, particularly through Th17 cell differentiation and proteoglycans in cancer pathways. Notably, ICOS is the target gene of lnc-ICOSLG-1, which is also referred to as LOC105372832. Based on this association, it is plausible to infer that lnc-ICOSLG-1 affects sepsis immunosuppression, specifically through the Th17 cell differentiation and proteoglycans in cancer pathways.

IKZF2 may play an important role in sepsis immunosuppression through the cytokine receptor interaction pathway. Deleting the IKZF2 gene results in immunodeficiency with dysregulated germinal center reactions by increasing the production of cytokines in Treg cells [30].

The current study has some limitations. For example, the number of samples was small. Nevertheless, the use of WGCNA was particularly advantageous for processing the gene expression datasets [31], and the results were validated by qPCR.

Another constraint of the study was the absence of validation by conventional biological experiments. Although our discoveries offer meaningful perspectives on the possible roles of these lncRNAs in sepsis immunosuppression, it is essential to corroborate the results through more direct experimental methods, including in vitro and in vivo tests.

## Conclusions

In summary, we identified Th17 cell differentiation (ko04659), proteoglycans in cancer (ko0520), and cytokine–cytokine receptor interaction (ko04060) pathways, as well as three essential lncRNAs (ENSG00000267074, lnc-ICOSLG-1, and lnc-IKZF2-7) correlated with sepsis immunosuppression. They may serve as biomarkers and/or targets for the diagnosis and treatment of sepsis immunosuppression.

## Supplementary Information

Supplementary Material 1.

## Data Availability

The dataset generated in this study has been submitted to Gene Expression Omnibus (GEO) database with accession number GSE286082.
