# Supplementary material for: Identification of lncRNAs in peripheral blood mononuclear cells associated with sepsis immunosuppression based on weighted gene co-expression network analysis
Source: Hereditas. 2025 Apr 7;162:51. doi: 10.1186/s41065-025-00400-z (PMC11974007; doi:10.1186/s41065-025-00400-z)
Supplement: Supplementary file 1 — Supplementary Material 1. [file 41065_2025_400_MOESM1_ESM.docx]

Supplementary Table 1

Descriptive statistics of sepsis immunosuppression group versus control group participants

|  | Sepsis Group (n=23) | Control Group (n=13) |
| --- | --- | --- |
| Age (years) | Mean (SD) | Mean (SD) |
|  | 49.6 (19.4) | 53.5 (18.7) |
|  |  |  |
| Sex | No. (%) | No. (%) |
| Male | 10 (43.5) | 5 (38.5) |
| Female | 13 (56.5) | 8 (61.5) |

Supplementary Table 2

| Number of lncRNAs in different modules | |
| --- | --- |
| module | number |
| blue | 1248 |
| brown | 819 |
| lightcyan | 78 |
| grey60 | 14 |
| salmon | 19 |
| cyan | 234 |
| tan | 13 |
| turquoise | 546 |
| red | 231 |
| yellow | 702 |
| greenyellow | 10 |
| pink | 155 |
| green | 56 |
| magenta | 24 |
| purple | 21 |
| black | 11 |
| midnightblue | 12 |

Supplementary Table 3

| The MM and GS value of Top 20 lncRNAs | | |
| --- | --- | --- |
| lncRNAs | MM | GS |
| lnc-NR1D1-1 | 0.999629691 | 0.975465491 |
| lnc-COPG1-1 | 0.999170816 | 0.975550164 |
| NONHSAG091824.1 | 0.998842677 | 0.962499356 |
| ENSG00000272950.1 | 0.998360395 | 0.974789402 |
| lnc-GIPR-1 | 0.998222894 | 0.970938656 |
| G085577 | 0.99810231 | 0.984849927 |
| lnc-MAP3K13-7 | 0.997401325 | 0.967564378 |
| lnc-LBH-3 | 0.997400744 | 0.967117979 |
| lnc-MTRNR2L8-3 | 0.997296991 | 0.964423126 |
| lnc-KMT2C-1 | 0.997254106 | 0.959042407 |
| NONHSAG063985.1 | 0.997240984 | 0.982101491 |
| lnc-PLAA-2 | 0.997234348 | 0.957632976 |
| NONHSAG074420.1 | 0.997084914 | 0.960815585 |
| ENSG00000286453.1 | 0.997013505 | 0.971319243 |
| NONHSAG100063.1 | 0.996805795 | 0.983758177 |
| lnc-STT3B-3 | 0.9965683 | 0.957524586 |
| G063788 | 0.996320588 | 0.976226423 |
| lnc-IKZF2-7 | 0.996275735 | 0.969274299 |
| ENSG00000267074 | 0.996043484 | 0.980739835 |
| lnc-ICOSLG-1 | 0.996016812 | 0.969133903 |

Supplementary Table 4

| The 64 target genes of the top 20 LncRNAs |
| --- |
| \| GATA2 \| CPA3 \| HBEGF \| HS3ST3 \| \| --- \| --- \| --- \| --- \| \| LAD1 \| CLEC4C \| PLAT \| RYK \| \| EIF4E \| TRBV4-2 \| IL2RG \| CD275 \| \| IL1R1 \| ICOSLG \| NRG2 \| UBD \| \| COX10 \| IL5 \| B7RP1 \|  \| \| PMCH \| SMKR1 \| IL15 \|  \| \| AP2B1 \| ANXA2 \| EHF \|  \| \| ERBB4 \| BTF3 \| IL2 \|  \| \| ICOS \| DELEC1 \| NACA \|  \| \| ITGB2 \| AP2M1 \| PABPC1 \|  \| \| SLFN12 \| KLRC1 \| RAMP1 \|  \| \| TFPI \| DNMT3L \| WNT5A \|  \| \| DNASE1L3 \| ICOSL \| ITPR2 \|  \| \| GRB2 \| GBP4 \| IL2RA \|  \| \| FAAH2 \| IKZF2 \| STAT5A \|  \| \| NDP \| MTOR \| IFNG \|  \| \| TNFSF15 \| HFE \| CALCRL \|  \| \| LIF \| NCCRP1 \| ANK3 \|  \| \| PTGDR2 \| HER4 \| SHC1 \|  \| \| FZD4 \| KCNK17 \| XCL1 \|  \| |

Supplementary Table 5

| Genes corresponding to top 15 pathways | | |
| --- | --- | --- |
| pathway | gene | p-value |
| Th17 cell differentiation | IL15, ICOSLG, ICOSL, CD275, SLFN12, IL2, IL5, ICOS, STAT5A, MTOR | 0.009069 |
| Proteoglycans in cancer | ITPR2, ERBB4, HER4, ICOS, DNMT3L, DELEC1, BTF3, ANK3 | 0.025529 |
| Cytokine-cytokine receptor interaction | TNFSF15, VEGI, IKZF2, IL1R1, IFNG | 0.030904 |
| Calcium signaling pathway | ITPR2, ERBB4, HER4, SEPHS2, ITGAL | 0.040747 |
| Glycosaminoglycan biosynthesis - heparan sulfate / heparin | HS3ST3, COX10 | 0.048958 |
| Long-term depression | IKZF2, ITPR2, IFNG | 0.052275 |
| Cortisol synthesis and secretion | CCL17, CXCR1 | 0.056772 |
| GnRH secretion | XCL1, IL1R1 | 0.057894 |
| Long-term potentiation | ITPR2, IL2RG | 0.060132 |
| Renin secretion | IFNG, CR1, ITPR2 | 0.064595 |
| Thyroid hormone synthesis | XCL1, HFE | 0.065708 |
| Gastric acid secretion | NDP, PMCH | 0.069039 |
| PI3K-Akt signaling pathway | EIF4E, ERBB4, HER4, AP2B1, SLFN12 | 0.070514 |
| EGFR tyrosine kinase inhibitor resistance | EIF4E, IL2 | 0.070786 |
| ErbB signaling pathway | ERBB4, HER4 | 0.071158 |

Supplementary Table 6

| The degree of the top 10 target genes | | | |
| --- | --- | --- | --- |
| node name | node degree | |  |
| SLFN12 | | 23 |  |
| ICOS | | 20 |  |
| IKZF2 | | 19 |  |
| GRB2 | | 15 |  |
| IL2 | | 15 |  |
| SHC1 | | 15 |  |
| IL2RA | | 14 |  |
| MTOR | | 14 |  |
| STAT5A | | 13 |  |
| HBEGF | | 13 |  |

Supplementary Table 7

| Gene ID | lncRNA ID | lncRNA Name | Pvalue | Log2FoldChange |
| --- | --- | --- | --- | --- |
| ENSG00000214999 | ENST00000399413 | ALOX12-AS1 | 0.098718 | 1.15897 |
| ENSG00000225542 | ENST00000662195 | ZNF385D-AS1 | 0.10388 | 1.534066 |
| ENSG00000230647 | ENST00000436469 | KLHL7-AS1 | 0.072366 | 0.823868 |
| ENSG00000249307 | ENST00000669293 | LINC01088 | 0.185641 | 0.276781 |
| ENST00000658400 | ENST00000592908 | UXT-AS1 | 0.103137 | 0.739282 |
| ENSG00000270673 | ENST00000604818 | IKZF2-210-AS | 0.092759 | 0.915446 |
| ENSG00000272382 | ENST00000607830 | ANXA2R-AS1 | 0.094243 | 0.768163 |
| ENSG00000272953 | ENST00000609130 | Linc02983 | 0.126809 | 0.410217 |
| ENSG00000273448 | ENST00000609770 | STAG3L4-203 | 0.319 | -0.19768 |
| ENSG00000277386 | ENST00000615722 | ZC3H7A-201 | 0.135242 | 0.792233 |

LncRNAs in turquoise module be validated in GSE201958

Supplementary Table 8

| Gene ID | Gene Symbol | Pvalue | Log2FoldChange |
| --- | --- | --- | --- |
| ENSG00000172123 | SLFN12 | 0.458049 | 0.05811 |
| ENSG00000030419 | IKZF2 | 0.901499 | -0.01162 |
| ENSG00000163600 | ICOS | 0.432091 | -0.056 |

Three hub genes be validated in GSE201958

Supplementary Table 9

| Gene Symbol | Forward primer | Reverse primer |
| --- | --- | --- |
| ENSG00000267074 | GCCTGTGGATTTCATTTCTGCCTTT | TTCTGAGTCTGCGTCCAGTTAGTATG |
| Lnc-IKZF2-7 | ACATGAGGCGAGGATAAAGTGGAGA | GCAAGGGCTCTGTAAGGCTTCC |
| Lnc-ICOSLG-1 | CCATCCAATGCCATAACCACAGACA | ACGAGCAGGTAGACGGTAAGAGG |
| SLFN12 | TTCTGCTGTGCAGTGTTTGC | TGCCGTTGCCATTCCAAAAG |
| IKZF2 | TGGCCCCATCTCTCTCATCA | ACTGGTGGTCATCATGGCTG |
| ICOS | CTTTCTCTTCTGCTTGCGCATT | AGATCGCAGAGTATTTGCCCC |
| GAPDH | TCAAGAAGGTGGTGAAGCAGG | GCGTCAAAGGTGGAGGAGTG |

The primers of hub genes for RT-PCR
